# The Human Factor: How Staff Attitudes Shape Mobility in Hospitalized Older Adults

**DOI:** 10.1093/geroni/igaf122.2194

**Published:** 2025-12-31

**Authors:** Anna Zisberg, Ksenya Shulyaev, Nurit Gur-Yaish, Juliana Smichenko, Efrat Shadmi

**Affiliations:** University of Haifa, Haifa, HaZafon, Israel; University of Haifa, Haifa, HaZafon, Israel; University of Haifa, Haifa, HaZafon, Israel; University of Haifa, Haifa, HaZafon, Israel; University of Haifa, Haifa, HaZafon, Israel

## Abstract

In-hospital mobility is crucial for older adults’ recovery, post-hospital function, and quality of life. While patient characteristics influence mobility, the impact of organizational factors, specifically unit workload and staff attitudes, remains underexplored. This study examined their effects on mobility among hospitalized older adults. We analyzed mobility data from 610 cognitively intact, ambulatory older adults (mean age 77.6±6.8, 54.1% male) across 20 units in four Israeli hospitals. Mobility was self-reported daily (up to 7 days) using the Mobility Rating (MR). Unit workload was assessed via nurse-to-patient ratio, patient turnover, and ventilated patient count. Staff attitudes (nurses, nursing assistants, physical therapists, and physicians) were measured using the Barriers to Early Mobility questionnaire. Covariates included age, pre-hospital ADL, cognitive status (MMSE), and acute illness severity (NEWS). Mixed-model analysis (5306 observations, 1079 data points) revealed that positive staff attitudes (β = 2.16, p = 0.046), higher ADL (β = 0.078, p < 0.0001), and better cognition (β = 0.099, p = 0.027) were associated with greater mobility. Conversely, older age (β = -0.06, p = 0.002) and higher acute illness severity (β = -0.211, p = 0.0012) predicted lower mobility. Notably, workload factors were not significantly associated with daily mobility. Findings highlight the critical role of staff attitudes in preserving mobility during hospitalization. Promoting a mobility-supportive hospital culture may be key to improving outcomes for older adults.

